# 低氧与肺癌的研究进展

**DOI:** 10.3779/j.issn.1009-3419.2013.04.08

**Published:** 2013-04-20

**Affiliations:** 300052 天津，天津市肺癌转移与肿瘤微环境重点实验室，天津市肺癌研究所，天津医科大学总医院 Tianjin Key Laboratory of Lung Cancer Metastasis and Tumor Microenvironment, Tianjin Lung Cancer Institute, Tianjin Medical University General Hospital, Tianjin 300052, China

**Keywords:** 低氧, 肺肿瘤, 低氧诱导因子, 脯氨酸羟化酶, 希佩尔•林道病基因产物, Hypoxia, Lung neoplasms, Hypoxia inducible factor, Prolyl hydroxylase domain, Product of von Hippel-Lindau gene

## Abstract

肺癌是我国发病率和死亡率增长最快, 对人群健康和生命威胁最大的恶性肿瘤, 其发生发展机制尚未完全清楚。肿瘤的低氧微环境发现于1955年, 而肺癌组织低氧直至2006年才被成功检测到。随着研究的深入, 低氧对肺癌的影响不仅限于对放疗的抵抗作用, 而且还会通过一个重要的促癌分子低氧诱导因子(hypoxia inducible factor, HIF)以及其调节蛋白脯氨酸羟化酶(prolyl hydroxylase domain, PHD)和希佩尔•林道病基因产物(product of von Hippel-Lindau gene, pVHL)对肺癌的发生发展、侵袭转移、化疗耐药以及预后等产生重要的调节作用。因此, 低氧、HIF、PHD和pVHL必将成为十分有潜力的肺癌治疗靶点。

## Abstract (en)

Lung cancer is one of the malignant tumors with fastest growing rates in incidence and mortality in our country, also with largest threat to human health and life.However, the exact mechanisms underlying lung cancer development remain unclear.The microenvironment of tumor hypoxia was discovered in 1955, but hypoxia in lung cancer tissues had not been successfully detected till 2006.Further studies show that hypoxia not only functions through the resistance to radiotherapy, but also regulates lung cancer development, invasion, metastasis, chemotherapy resistance and prognosis through an important oncogene HIF (hypoxia inducible factor), with its regulators PHD (prolyl hydroxylase domain) and pVHL (product of von Hippel-Lindau gene).Therefore, hypoxia, HIF, PHD and pVHL should be considered as potential therapeutic targets for lung cancer pathogenesis and progression.

肺癌是发病率和死亡率增长最快, 对人群健康和生命威胁最大的肿瘤。全球每年大约有160万的肺癌新发病例和140万的肺癌死亡病例^[1]^。在美国肺癌的发病率在所有恶性肿瘤中位列第2位, 而死亡率列第1位^[2]^。根据卫生部2012年度肿瘤登记年报资料^[3]^显示, 我国2009年肺癌发病率为53.57/10万, 死亡率为45.57/10万, 即每年新发肺癌患者超过70万, 死亡超过60万。

已有的研究^[4, 5]^表明, 肺癌发病原因与多种基因的突变密切相关, 其中最为重要和典型的是抑癌基因p53的突变和原癌基因K-ras的突变。此外, 约有40%-50%的肺腺癌中存在表皮生长因子受体(epidermal growth factor receptor, EGFR)的异常高表达或激活突变, 并且与较差的预后相关联^[6, 7]^。与非吸烟人群相比, p53和K-ras的突变比例在吸烟人群中略高; 而EGFR的突变比例在非吸烟人群中远高于吸烟人群^[8]^。因此, 以这些基因作为靶点寻找治疗肺癌的方法具有很强的可行性。此外, 还有一种重要的肿瘤微环境因素-低氧(hypoxia), 对肺癌的发生发展具有重要的作用。因此, 本文将近年来低氧与肺癌相关性的研究进展综述如下。

## 1 低氧的概念

肿瘤是非单一细胞的实体, 从总体上看包括肿瘤细胞和间质, 而间质又主要包括血管、淋巴管、结缔组织、炎症细胞和细胞外基质等。此外, 可溶性分子O_2_还参与肿瘤细胞附近的间质动态变化, 促进肿瘤的恶性化^[9]^, 他们共同组成了肿瘤的微环境。机体各组织器官的氧分压明显低于标准大气压160 mmHg, 且各器官并不同, 其中由高到低分别为:肺110 mmHg, 脾66 mmHg, 心25 mmHg, 肾25 mmHg, 脑24 mmHg, 肝24 mmHg^[10]^。低氧是指可利用氧的减少或氧分压降至临界值以下的状态, 这种状态会限制甚至终止器官、组织和细胞的生理功能^[11]^。低氧可以由多种因素造成^[11]^, 而低氧常发生在急慢性血管性疾病、肺部疾病和肿瘤中^[12]^。

## 2 肺癌低氧微环境的发现

其实, 早在50多年前, Thomlinson和Gray^[13]^就在距血管180 μm的地方观察到了肿瘤坏死, 他们推测这是肿瘤细胞过度增殖使其将血氧完全耗尽的位置, 这是人类首次开创性地观测并推断了肿瘤低氧微环境的存在。此后, Höckel等^[14]^也利用氧电极在头颈部、颈椎肿瘤和乳腺癌中发现了低氧存在, 并且与肿瘤患者的低存活率和肿瘤的高转移性均具有相关性。后续的研究^[15]^发现, 放疗过程中氧气可以被激发而产生自由基, 杀伤肿瘤细胞, 但处于低氧区的肿瘤细胞由于缺乏氧气, 自由基产生较少, 从而对放疗产生抵抗作用。直到2006年, Le等^[16]^才在肺癌患者体内检测到了肺癌组织低氧气分压的存在, 并且与较差的预后相关, 这是迄今为止唯一关于检测到肺癌组织低氧的报道。

## 3 低氧的信号通路

随着研究的深入, 人们发现低氧会通过一个能够感受并应对低氧的重要转录因子-低氧诱导因子(hypoxia inducible factor, HIF), 对肿瘤产生多方面的影响。HIF是由Semenza等^[17]^在研究低氧加速红细胞成熟时发现的。它是一个由受氧调节的α亚基与稳定表达的β亚基组成的异源二聚体。在常氧条件下, HIF-α蛋白上两个关键位置的脯氨酸会被脯氨酸羟化酶(prolyl hydroxylase domain, PHD)羟化, 使其可被希佩尔•林道病基因产物(product of von Hippel-Lindau gene, pVHL)识别并结合, 进而被pVHL降解复合物泛素化降解; 而在低氧条件下, PHD活性受到抑制, HIF-α无法被羟基化, 从而无法被pVHL识别、结合并介导降解, HIF-α便会大量积累, 结合HIF-1β, 入核发挥转录因子的功能, 在肿瘤的血管生成、细胞生存、增殖、凋亡、转移、浸润和代谢等方面发挥重要的作用。而在2009年HIF也被发现在肺癌组织中转录水平上高表达, 并且与较差的预后相关联^[18]^。此后, 越来越多的研究^[19]^证明低氧和HIF有可能成为治疗肺癌的一个十分重要的靶点。

## 4 低氧信号通路对肺癌的作用

低氧可以通过减少自由基产生, 从而对肺癌的放疗产生抵抗作用。而低氧对肺癌的影响远不止这些, Minakata等^[20]^发现在*EGFR*突变的非小细胞肺癌中, 低氧可以通过上调转化生长因子(transforming growth factor alpha, TGF-α)来激活野生型EGFR, 从而使肺癌细胞产生对吉非替尼的耐药性; Ouyang等^[21]^还发现低氧可以上调骨膜蛋白(periostin)的表达, 通过激酶Akt/PKB信号通路提高肺癌细胞的生存能力。其实, 低氧相关信号分子如低氧诱导因子HIF、脯氨酸羟化酶PHD和希佩尔•林道病基因产物pVHL均会通过多条途径对肺癌的发生发展产生影响而发挥更加重要的作用。

### 4.1 HIF对肺癌的作用

HIF由α和β两个亚基组成。在哺乳动物中, HIF-α存在三种亚型, HIF-1α和HIF-2α在结构和功能上相似性较大, 而HIF-3α研究较少, 有报道^[22]^认为它可能作为HIF-1α和HIF-2α的负调控元件。HIF-1α在几乎所有类型的细胞中均有表达, 而HIF-2α和HIF-3α的表达则具有一定的组织局限性^[23]^。研究表明, 低氧可以通过HIF-1α或HIF-2α对肺癌产生重要影响。

#### 4.1.1 HIF在肺癌组织中高表达

早在1999年Zhong等^[24]^通过大量的免疫组化染色实验发现, HIF-1α在19种肿瘤的13种中呈现高表达, 其中包括肺癌。2000年Volm等^[25]^在96例非小细胞肺癌标本中对HIF-1α和HIF-1β进行了免疫组化染色, 他们发现HIF-1阳性患者的平均存活时间明显高于HIF-1阴性患者, 提示与较好的预后有关联。2001年Giatromanolaki等^[26]^应用免疫组化染色对HIF-1α或HIF-2α进行的检测, 结果显示二者分别在62%和50%的非小细胞肺癌病例中高表达, 并且与多种血管生长因子的表达呈正相关。但与之前的报道相反, 他们发现HIF-1α与较差的预后相关联。2009年Yohena等^[18]^通过实时荧光定量PCR手段在肺癌组织中发现了HIF-1α在转录水平上的高表达, 这与之前报道的蛋白水平结果相一致, 同时他们也发现HIF-1α与较差的预后密切相关。

#### 4.1.2 HIF对肺癌细胞增殖和凋亡的影响

2006年Zhang等^[27]^在肺腺癌A549细胞中外源性地表达了HIF-1α, 他们发现, 与对照细胞相比表达HIF-1α的A549细胞可以通过上调多药耐药基因*MDR-1*, 抵抗由5-氟尿嘧啶诱导的细胞凋亡。Kamlah等^[28]^在LLC1肺腺癌模型小鼠中, 利用颈静脉siRNA注射技术, 同时沉默HIF-1α和HIF-2α的表达, 发现进行基因沉默的肺癌模型小鼠存活时间明显增长, 并且免疫组化显示肿瘤的增殖明显减慢, 血管生成和凋亡也都明显减少。同年, Chen等^[29]^从大量的非小细胞肺癌组织标本出发, 发现了HIF-1α与凋亡抑制蛋白(inhibitor of apoptosis protein, IAP)家族成员蛋白survivin表达的正相关性, 并在细胞实验中发现, HIF-1α可以在转录水平调节survivin的表达, 从而抑制肺癌细胞的凋亡。HIF-1α在小细胞肺癌中的作用是由Wan等^[30]^报道, 他们通过直接低氧、基因过表达和基因沉默三种手段改变HIF-1α的表达, 并进行基因表达谱分析, 发现HIF-1α可以促进细胞因子信号传导抑制蛋白-1(suppressor of cytokine signaling, SOCS1)基因的表达, 而SOCS1可以促进小细胞肺癌NCI-H446细胞的凋亡并抑制其生长。这一结果首次揭示了HIF-1α在小细胞肺癌中的特殊调节机制, 即通过*SOCS1*基因对自身的促癌作用进行负反馈调节。Mazumdar等^[31]^也利用K-ras(G12D)肺癌小鼠模型, 揭示了HIF-2α的两面作用, 他们提出HIF-2α不仅能够促进肺癌的发展, 还能通过调节抑癌基因, 发挥抑制肺癌的作用。以上这些报道表明, HIF在低氧加速肺癌进程的过程中发挥着十分重要的作用。

#### 4.1.3 HIF在肺癌细胞侵袭转移中的作用

2007年Shyu等^[32]^在利用不同侵袭能力的配对肺癌细胞系CL1和CL1-5研究HIF-1α的表达时发现, HIF-1α仅在高侵袭能力的CL1-5细胞系中表达, 并且沉默HIF-1α表达后可以明显降低其侵袭能力。深入的研究发现, HIF-1α可以通过上调基质金属蛋白酶(matrix metalloproteinase, MMP)MMP1和MMP2的表达来促进肺癌细胞的侵袭。Li等^[33]^在2009年利用94例非小细胞肺癌标本进行免疫组化染色分析, 发现HIF-1α、HIF-2α与CC趋化因子受体7(CC chemokine receptor 7, CCR7)的表达呈现正相关性, 而深入的细胞实验结果显示, HIF-1α和HIF-2α可以正向调节CCR7的表达, 而CCR7又可以通过促进激酶ERK的磷酸化来促进肺癌细胞的迁移。2010年Jacoby等^[34]^利用两株非小细胞肺癌细胞模型和两株小细胞肺癌细胞模型, 在裸鼠中进行了实验, 发现用HIF-1α的拮抗剂PX-478对小鼠进行灌胃处理, 可以明显抑制原位肿瘤的生长和纵隔转移, 并与较好的预后相关。以上研究结果均表明, 低氧可以通过HIF促进肺癌侵袭转移, 从而影响肺癌的预后。

### 4.2 PHD在肺癌中的作用

脯氨酸羟化酶PHD家族有PHD1、PHD2和PHD3三个成员, 在低氧和常氧条件下发挥降低抑癌蛋白HIF的作用。2006年Davidson等^[35]^在肺腺癌A549细胞中发现, 可溶性的镍可以抑制PHD的羟化功能以及PHD与HIF的作用, 从而稳定了HIF, 此项研究开创性地提出PHD可以作为肺癌治疗的靶点。2008年Giatromanolaki等^[36]^利用73例非小细胞肺癌患者标本进行研究发现, 其中的33例标本具有高HIF/PHD表达, 而只有18例标本具有低的HIF/PHD表达, 这一结果在临床标本水平提示了HIF的促癌作用和PHD的抑癌作用。2001年Chen等^[37]^发现调节HIF的关键分子脯氨酸羟化酶PHD1、PHD2和PHD3均在62例配对非小细胞肺癌标本的癌组织中高表达。通过与临床资料和病理结果的相关性分析, 发现PHD3的高表达与肿瘤分期较早及分化相关, 通过病理切片染色还发现PHD3的高表达与抗凋亡蛋白Bcl-2呈负相关。这一报道提示在非小细胞肺癌中PHD3可能具有潜在的促凋亡功能。2011年Andersen等^[38]^扩大了样本量, 他们利用335例Ⅰ期到Ⅲ期的非小细胞肺癌标本进行组织芯片和免疫组化染色实验, 检测了三种脯氨酸羟化酶的表达情况, 并分析与预后的关系。研究结果显示PHD1、PHD2和PHD3在肺癌标本中均高表达, 并且与较差的预后相关联, 具有高表达PHD的病例5年存活率明显低于低表达组。这一结果提示脯氨酸羟化酶也可能具有促癌作用。在2012年最新的一项研究中, Chen等^[39]^应用原位杂交技术和免疫组化染色技术, 在26例肺癌标本中检测了HIF及三种脯氨酸羟化酶的表达情况, 其中12例病例伴有慢性阻塞性肺疾患(chronic obstructive pulmonary disease, COPD), 而其余14例为对照组。通过医学统计学分析得出结论, 脯氨酸羟化酶通过调节HIF, 在肺癌病例中参与了COPD的低氧肺血管重建过程。

### 4.3 pVHL在肺癌中的作用

虽然pVHL被证明可以通过泛素化降解途径下调促癌蛋白HIF而发挥抑癌基因的功能。但是, 肺癌方面功能的报道中却显示pVHL存在两方面作用。1997年Corless等^[40]^率先通过免疫组化染色发现, pVHL主要在肺癌细胞的细胞质有较强的表达。2001年Kamada等^[41]^发现肺癌H1299细胞中导入外源表达的pVHL, 可以稳定肌动蛋白组织装配, 并通过促进黏着斑形成, 抑制肺癌细胞的迁移能力, 这是首次发现pVHL在肺癌细胞中具有抑癌作用。而最新的研究报道显示了pVHL的促癌作用。Zhou等^[42]^的研究发现, 在肺腺癌细胞A549中稳定沉默pVHL表达可以明显降低细胞的成克隆能力和增殖能力, 揭示了pVHL同样可以发挥促癌作用, 并且暗示了非HIF依赖的pVHL新功能。

## 5 结语

从肿瘤低氧微环境最初被发现到现在的60年中, 人们已经清楚地了解了它的存在、调节机制和意义及功能。越来越多的研究表明, 低氧对各种类型的肿瘤包括肺癌均起到十分重要的作用。低氧诱导因子HIF也已成为肺癌发生发展过程中一个极其重要的调控因子。HIF在肺癌中的高表达与增殖和预后等相关联, 使其可以作为肺癌发生发展和预后的分子标记存在。此外, 调节HIF的重要分子PHD和pVHL也在肺癌组织细胞中主要呈现低表达, 并发挥重要的功能。这使得低氧、低氧诱导因子以及其调节分子PHD和pVHL成为十分有潜力的肺癌治疗靶点。
